# Microarray Based Gene Expression Analysis of Murine Brown and Subcutaneous Adipose Tissue: Significance with Human

**DOI:** 10.1371/journal.pone.0127701

**Published:** 2015-05-26

**Authors:** Ritesh K. Baboota, Siddhartha M. Sarma, Ravneet K. Boparai, Kanthi Kiran Kondepudi, Shrikant Mantri, Mahendra Bishnoi

**Affiliations:** 1 National Agri-Food Biotechnology Institute (NABI), SAS Nagar, Punjab, India; 2 Department of Biochemistry, Panjab University, Chandigarh, India; The Perinatal Institute, Cincinnati Children's Hospital Medical Center and University of Cincinnati, UNITED STATES

## Abstract

**Background:**

Two types of adipose tissues, white (WAT) and brown (BAT) are found in mammals. Increasingly novel strategies are being proposed for the treatment of obesity and its associated complications by altering amount and/or activity of BAT using mouse models.

**Methodology/Principle Findings:**

The present study was designed to: (a) investigate the differential expression of genes in LACA mice subcutaneous WAT (sWAT) and BAT using mouse DNA microarray, (b) to compare mouse differential gene expression with previously published human data; to understand any inter- species differences between the two and (c) to make a comparative assessment with C57BL/6 mouse strain. In mouse microarray studies, over 7003, 1176 and 401 probe sets showed more than two-fold, five-fold and ten-fold change respectively in differential expression between murine BAT and WAT. Microarray data was validated using quantitative RT-PCR of key genes showing high expression in BAT (*Fabp3*, *Ucp1*, *Slc27a1*) and sWAT (*Ms4a1*, *H2-Ob*, *Bank1*) or showing relatively low expression in BAT (*Pgk1*, *Cox6b1*) and sWAT (*Slc20a1*, *Cd74*). Multi-omic pathway analysis was employed to understand possible links between the organisms. When murine two fold data was compared with published human BAT and sWAT data, 90 genes showed parallel differential expression in both mouse and human. Out of these 90 genes, 46 showed same pattern of differential expression whereas the pattern was opposite for the remaining 44 genes. Based on our microarray results and its comparison with human data, we were able to identify genes (targets) (a) which can be studied in mouse model systems to extrapolate results to human (b) where caution should be exercised before extrapolation of murine data to human.

**Conclusion:**

Our study provides evidence for inter species (mouse vs human) differences in differential gene expression between sWAT and BAT. Critical understanding of this data may help in development of novel ways to engineer one form of adipose tissue to another using murine model with focus on human.

## Background

White (WAT) and brown (BAT) adipose tissue have different biological roles in mammals. WAT, which represents at least 10% of the total body weight of a normal healthy adult, is the main site for energy storage and acts as an endocrine organ that signals to other tissues through adipokines [1]. On the other hand, BAT is a specialized tissue for energy dissipation and thermoregulation. Rodent data has suggested the existence of two forms of BAT which are derived from different lineages and can affect the amount and presence of one another i.e. constitutive (myogenic lineage, embryonic origin and present in interscapular region of mice) and recruitable (non-myogenic lineage, present within WAT and skeletal muscle) BAT [2–4]. The latter are also known as “brite”/”beige” adipocytes and have many of the morphological and physiological characteristics of classical BAT [4–6]. Recent reports have confirmed the presence and activity of BAT in human adults [7–10]. In humans, BAT depots are mainly observed in the supraclavicular, paravertebral regions and in the neck and express high levels of *PRDM16*, *PGC-1α* and *UCP-1* [7–9,11–15]. Using 18F-fluorodeoxyglucose (FDG) positron emission tomography (PET) combined with X-ray CT, these studies have clarified conditions in humans which have correlated with activated (increase in circulating catecholamines and cold exposure) and inactivated (obesity) BAT [8,14]. Several independent reports have shown the presence of functional BAT present within WAT, especially near vertebrae and clavicles [13,16]. Based on the amount of activated BAT in these studies and its direct relation with thermogenesis and energy dissipation, it is now certain that these tissues play a significant and critical role in whole body energy homeostasis. Further, analysis of large number of FDG-PET scans suggests an inverse correlation between BAT volume and body mass index [11,12,14]. Independent of their origin and location, brite cells and the stimulation of WAT *“browning”* could be useful in the fight against obesity and related complications. Presently, molecular mechanisms involved in WAT *“browning”* and role of phytochemicals/dietary constituents in this phenomenon is an active area of research and has opened opportunities for the development of novel strategies for the treatment of obesity and its associated complications.

The conversion of WAT into BAT/“brite” is a promising strategy but translation of rodent data to human is a challenge. A recent report by Svensson *et al* and other studies, have shown that there are species-specific differences in BAT gene expression and suggests the need for in-depth molecular characterization of human BAT to have better understanding of genes, pathways and mechanisms involved in formation of BAT and its conversion into WAT in humans. A comparison between mouse and human differential gene expression in WAT and BAT is needed so that extrapolation and translation of mouse data to human can be done. The present study was undertaken to investigate the differential gene expression in murine (LACA) sWAT and BAT using DNA microarray studies to identify novel genes and pathways that can be targeted for converting WAT to BAT or increase the amount of BAT in WAT. Further, using available gene expression data of human WAT and BAT [17,18] we have compared murine differential expression with human differential expression to identify genes (targets) (a) which can be studied in mouse model systems to extrapolate the results to human (b) where caution should be exercised before extrapolation of murine data to human.

## Results

### Microarray analysis of gene expression profiles in sWAT and BAT

Upon comparing the gene expression profile of murine sWAT and BAT using the criteria described in materials and methods section, of the 45101 probe sets present on the mouse genome array we identified 7003, 1176 and 401 probes showing greater than two-fold, five-fold and ten-fold change respectively which showed significant differential expression between sWAT and BAT (S1 Table). Out of 7003 differentially expressed probe sets, 38.80% (2717) were highly expressed in BAT as compared to sWAT and remaining 61.20%(4286) showed higher expression in sWAT as compared to BAT. Of the 401 probe sets with ten-fold change, 113 were up-regulated in BAT and the rest were up-regulated in sWAT.

Top Differentially expressed genes in murine BAT include myosin heavy polypeptide 1 (Mm.422801) and uncoupling protein 1(Mm.4177) among several others (Table 1). Top differentially expressed genes in murine sWAT include glycosylation dependent cell adhesion molecule 1 (Mm.219621), immunoglobulin heavy variable 14–2 (Mm.491093), immunoglobulin heavy constant mu (Mm.342177), FAS apoptotic inhibitory molecule 3 (Mm.46042) and DEAD box poly peptide 3 (Mm.446787), etc (Table 2).

**Table 1:** Top 10 genes highly expressed in Murine BAT.

S.no	Gene Name	Gene symbol	FC (Abs)	Unigene
1	Myosin, heavy polypeptide 1	Myh1	279.95	Mm.422801
2	Uncoupling protein 1	Ucp1	190.85	Mm.4177
3	Fatty acid binding protein 3	Fabp3	168.49	Mm.388886
4	Solute carrier family 27 (fatty acid transporter), member 2	Slc27a2	130.20	Mm.290044
5	Troponin C2, fast	Tnnc2	88.44	Mm.1716
6	Myotilin	Myot	83.41	Mm.143804
7	Myosin, heavy polypeptide 4	Myh4	74.78	Mm.297382
8	Actin, alpha 1, skeletal muscle	Acta1	74.09	Mm.214950
9	Cytochrome c oxidase subunit VIIa 1	Cox7a1	69.90	Mm.423030
10	Troponin T3, skeletal, fast	Tnnt3	61.26	Mm.389992

Genes differentially expressed in murine BAT along with absolute fold changes.

**Table 2:** Top 10 genes highly expressed in Murine WAT.

S.no	Gene Name	Gene symbol	FC (Abs)	Unigene
1	Glycosylation dependent cell adhesion molecule 1	Glycam1	240.14	Mm.219621
2	Immunoglobulin heavy variable 14–2	Ighv14-2	179.35	Mm.491093
3	Histocompatibility 2, O region beta locus	H2-Ob	131.83	Mm.327358
4	Immunoglobulin heavy constant mu	Igh-6	121.17	Mm.342177
5	Membrane-spanning 4-domains, subfamily A, member 1	Ms4a1	97.19	Mm.4046
6	Immunoglobulin heavy constant gamma 1 (G1m marker)	Ighg1	87.36	-
7	Predicted gene 3579	Gm3579	59.07	-
8	Fas apoptotic inhibitory molecule 3	Faim3	56.04	Mm.46042
9	POU domain, class 2, associating factor 1	Pou2af1	55.16	Mm.897
10	DEAD (Asp-Glu-Ala-Asp) box polypeptide 3, Y-linked	Ddx3y	53.51	Mm.446787

Genes differentially expressed in murine subcutaneous WAT along with absolute fold changes.

### Comparison of differential gene expression (between sWAT and BAT) of mouse with human

**Fig 1:**
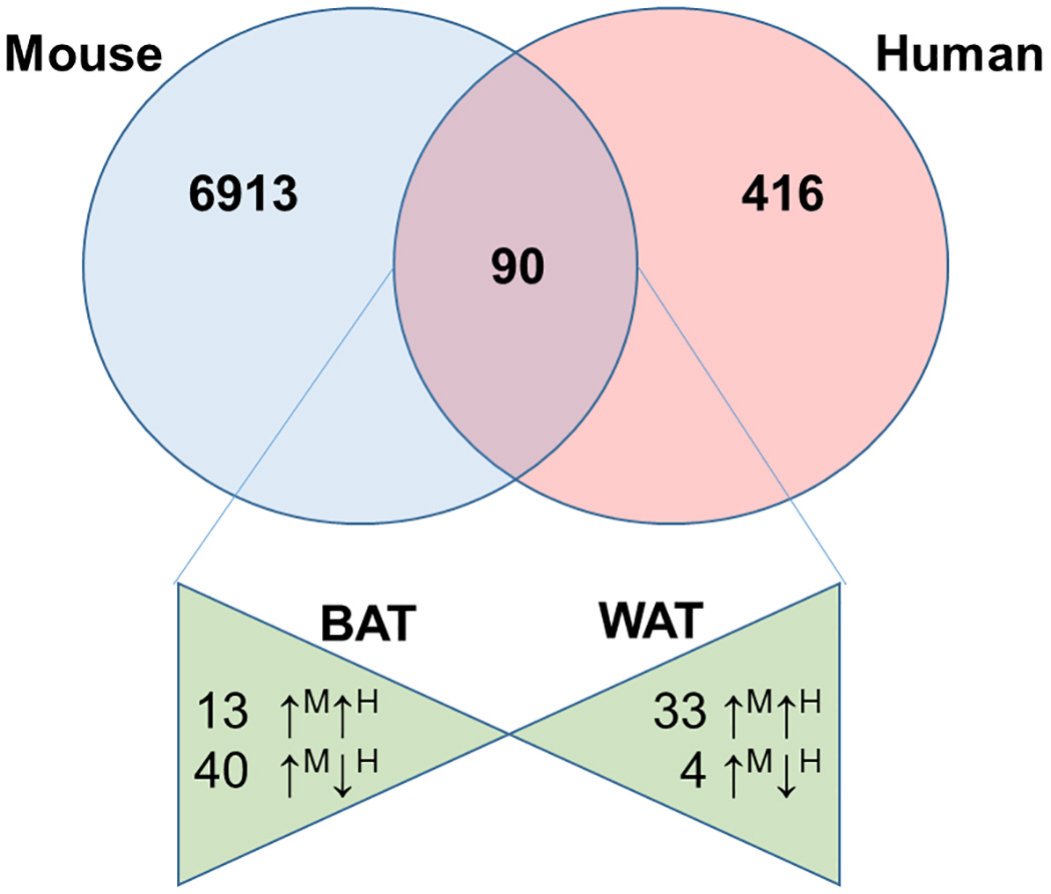
Venn diagram of greater than 2 fold expression of murine and human probe sets. Out of the common probes, the triangles represent the spread of same in both tissues with the arrows in text representing the similar or opposite expression in the two organisms. (Superscript: M—Mouse, H—Human). mRNA prepared and analyzed by Affymetrix microarray as described. All repeated probe sets of the same gene symbol were removed. Probe sets not updated with gene symbols were also removed. Differentially expressed genes were analyzed by volcano plots (Paired T Test, P≤0.05) generated from the list of 7003 significant genes selected by Paired T Test (P≤0.05) using Genespring GX 12.1.

Mouse is a key mammalian model system for studying complex human diseases and therefore it is necessary to investigate both the differences and similarities between mouse and human gene patterns, before extrapolation from mouse models to human systems. Hence, our study aims to investigate the interspecies (mouse vs. human) variation in gene expression between sWAT and BAT of human and mouse. First, we analyzed the already available microarray data of human WAT and BAT to identify their differential gene expression[17]. It was then compared with our murine data. The data submitted by Svensson et. al. (GSE27657), when analyzed showed 506 probe sets having ≥2fold change, 30 probe sets having ≥5 fold change and 10 probe sets having ≥10 fold change (Data not included). Top 10 genes that were highly expressed in Human BAT (Table 3) and sWAT (Table 4) analyzed using the same statistical parameters are shown. Fig 1 represents a Venn diagram showing comparison between the two organisms. Comparative analysis of differentially regulated genes of WAT vs. BAT in human and mice based on gene symbols led to identification of 90 genes common to both. Of these 90, 46 genes had similar expression profile in both organisms, with 13 genes specifically expressed in BAT, such as *UCP1*, *FABP3*, etc. and 33 genes in WAT, such as *LEP*, *IGF1*, *ENNP1*, *BCL2* etc. (Tables 5 & 6). Interestingly, there were 44 genes that showed opposite pattern of differential expression such as *Kcnb1* (Mm.46181), *Irx5* (Mm.101153), *Bdh1* (Mm.293470), *Slc25a16*(Mm.374557) (Table 7).

**Table 3:** Top 10 genes highly expressed in Human BAT (Svensson et al., GSE27657).

S.no.	Gene Name	Gene symbol	FC (Abs)	Unigene
1	Uncoupling protein 1	UCP1	48.20	Hs.249211
2	Creatine kinase, mitochondrial 1A/1B	CKMT1A /// CKMT1B	12.33	Hs.425633
3	Potassium channel, subfamily K, member 3	KCNK3	8.39	Hs.645288
4	Cordon-bleu WH2 repeat protein	COBL	6.57	Hs.99141
5	Carbonic anhydrase XII	CA12	5.06	Hs.210995
6	Creatine kinase, mitochondrial 2 (sarcomeric)	CKMT2	4.66	Hs.80691
7	3-hydroxy-3-methylglutaryl-CoA synthase 2 (mitochondrial)	HMGCS2	4.39	Hs.59889
8	Cytoplasmic FMR1 interacting protein 2	CYFIP2	4.13	Hs.519702
9	Angiotensinogen (serpin peptidase inhibitor, clade A, member 8)	AGT	4.12	Hs.19383
10	Arginase 2	ARG2	4.01	Hs.723133

Genes differentially expressed in human BAT with absolute fold change.

**Table 4:** Top 10 genes highly expressed in human WAT (Svensson et. al., GSE27657).

S.no	Gene Name	Gene symbol	FC (Abs)	Unigene
1	EGF-like-domain, multiple 6	EGFL6	30.94	Hs.12844
2	Colorectal neoplasia differentially expressed (non-protein coding)	CRNDE	25.74	Hs.237396
3	Cyclin-dependent kinase inhibitor 2B (p15, inhibits CDK4)	CDKN2B	16.89	Hs.72901
4	Triadin	TRDN	12.50	Hs.654601
5	Iroquois homeobox 1	IRX1	12.00	Hs.424156
6	Collagen, type VIII, alpha 1	COL8A1	11.65	Hs.654548
7	Doublesex and mab-3 related transcription factor 2	DMRT2	11.32	Hs.59506
8	Arylacetamide deacetylase	AADAC	7.60	Hs.506908
9	Iroquois homeobox 2	IRX2	7.39	Hs.282089
10	Dickkopf WNT signaling pathway inhibitor 2	DKK2	7.35	Hs.211869

Genes differentially expressed in human WAT with absolute fold change.

**Table 5:** Common genes expressed in BAT of both organisms.

S. no.	Gene Symbol	Annotation	Unigene ID	Entrez Gene	Mouse Ratio (BAT/WAT)
1	aco2	Aconitase 2, mitochondrial	Mm.154581	11429	7.76
2	cisd1	CDGSH iron sulfur domain 1	Mm.254114	52637	4.42
3	ckmt2	Creatine kinase, mitochondrial 2	Mm.316438	76722	46.88
4	clstn3	Calsyntenin 3	Mm.193701	232370	3.91
5	ebf2	Early B cell factor 2	Mm.319947	13592	4.27
6	fabp3	Fatty acid binding protein 3, muscle and heart	Mm.388886	14077	168.49
7	kcnk3	Potassium channel, subfamily K, member 3	Mm.439936	16527	8.64
8	ppif	peptidylprolyl isomerase F (cyclophilin F)	Mm.41656	105675	2.83
9	pxmp2	peroxisomal membrane protein 2	Mm.21853	19301	2.21
10	rxrg	retinoid X receptor gamma	Mm.3475	20183	3.14
11	slc27a2	solute carrier family 27 (fatty acid transporter), member 2	Mm.290044	26458	130.20
12	ucp1	Uncoupling protein 1	Mm.4177	22227	190.85
13	wnt5a	wingless-related MMTV integration site 5A	Mm.287544	22418	2.35

Common genes that are up-regulated in BAT of human and LACA mouse along with absolute fold change.

**Table 6:** Common genes expressed in WAT of both organisms.

S. no.	Gene Symbol	Annotation	Unigene ID	Entrez Gene	Mouse Ratio (WAT/BAT)
1	adra2a	Adrenergic receptor, alpha 2a	Mm.235195	11551	2.37
2	agfg1	ArfGAP with FG repeats 1	Mm.392569	15463	2.66
3	arhgef6	Rac/Cdc42 guanine nucleotide exchange factor (GEF) 6	Mm.261443	73341	2.92
4	bcl2	B cell leukemia/lymphoma 2	Mm.257460	12043	15.40
5	ccnd2	Cyclin D2	Mm.333406	12444	4.97
6	clmn	Calmin	Mm.244078	94040	2.03
7	col6a3	Collagen, type VI, alpha 3	Mm.7562	12835	2.68
8	cpm	Carboxypeptidase M	Mm.339332	70574	2.58
9	cpxm1	Carboxypeptidase X 1 (M14 family)	Mm.112701	56264	3.54
10	dhfr	Dihydrofolate reductase	Mm.23695	13361	2.25
11	dnase1l3	Deoxyribonuclease 1-like 3	Mm.272258	13421	4.42
12	dpt	Dermatopontin	Mm.28935	56429	2.82
13	dsc2	Desmocollin 2	Mm.280547	13506	6.57
14	dse	Dermatan sulfate epimerase	Mm.34557	212898	3.58
15	enpp1	Ectonucleotide pyrophosphatase/phosphodiesterase 1	Mm.27254	18605	6.25
16	enpp2	Ectonucleotide pyrophosphatase/phosphodiesterase 2	Mm.250256	18606	2.28
17	fgf13	Fibroblast growth factor 13	Mm.7995	14168	4.82
18	fmod	Fibromodulin	Mm.287146	14264	5.53
19	gng2	Guanine nucleotide binding protein (G protein), gamma 2	Mm.41737	14702	3.42
20	igf1	Insulin-like growth factor 1	Mm.268521	16000	3.37
21	lair1	Leukocyte-associated Ig-like receptor 1	Mm.290880	52855	3.25
22	lep	Leptin	Mm.277072	16846	3.17
23	mbnl3	Muscleblind-like 3 (Drosophila)	Mm.295324	171170	4.89
24	nnat	Neuronatin	Mm.34330	18111	3.87
25	ogfrl1	Opioid growth factor receptor-like 1	Mm.28013	70155	4.24
26	pcdh7	Protocadherin 7	Mm.332387	54216	3.84
27	prr16	Proline rich 16	Mm.63546	71373	2.69
28	sec62	SEC62 homolog (S. cerevisiae)	Mm.26017	69276	2.53
29	shox2	Short stature homeobox 2	Mm.39093	20429	2.62
30	spon1	Spondin 1, (f-spondin) extracellular matrix protein	Mm.334160	233744	2.11
31	tns3	Tensin 3	Mm.337820	319939	2.86
32	vgll3	Vestigial like 3 (Drosophila)	Mm.25670	73569	2.84
33	wdr26	WD repeat domain 26	Mm.289082	226757	2.03

Common genes that are up-regulated in WAT of human and LACA mouse along with their absolute fold change.

**Table 7:** Common genes expressed in both species while showing opposite trend of expression.

S. no.	Gene Symbol	Annotation	Unigene ID	Entrez Gene	Ratio in mouse (WAT/ BAT)	Ratio in Human (WAT/ BAT)
**Genes down-regulated in murine WAT while up-regulated in human WAT**						
1	acvr1c	Activin A receptor, type IC	Mm.77751	269275	-2.59	2.32
2	adam12	A disintegrin and metallopeptidase domain 12 (meltrin alpha)	Mm.439714	11489	-2.21	3.19
3	adrbk2	Adrenergic receptor kinase, beta 2	Mm.285619	320129	-2.30	2.11
4	aig1	Androgen-induced 1	Mm.45755	66253	-2.87	2.09
5	arhgap21	Rho GTPase activating protein 21	Mm.28507	71435	-2.22	2.02
6	asph	Aspartate-beta-hydroxylase	Mm.222206	65973	-3.85	2.38
7	cap2	CAP, adenylate cyclase-associated protein, 2 (yeast)	Mm.44529	67252	-3.58	3.32
8	ccdc85a	Coiled-coil domain containing 85A	Mm.93759	216613	-3.29	2.14
9	col12a1	Collagen, type XII, alpha 1	Mm.3819	12816	-2.08	5.11
10	cryab	Crystallin, alpha B	Mm.178	12955	-2.50	2.61
11	cxcl14	Chemokine (C-X-C motif) ligand 14	Mm.30211	57266	-3.54	2.91
12	ddit4l	DNA-damage-inducible transcript 4-like	Mm.250841	73284	-4.66	2.01
13	dmd	Dystrophin, muscular dystrophy	Mm.275608	13405	-2.69	2.13
14	fhl1	Four and a half LIM domains 1	Mm.3126	14199	-4.77	2.26
15	fibin	Fin bud initiation factor homolog (zebrafish)	Mm.291809	67606	-2.68	2.25
16	fmo1	Flavin containing monooxygenase 1	Mm.976	14261	-4.88	2.79
17	hspb7	Heat shock protein family, member 7 (cardiovascular)	Mm.46181	29818	-4.94	3.71
18	irx5	Iroquois related homeobox 5 (Drosophila)	Mm.101153	54352	-2.97	4.86
19	kcnb1	Potassium voltage gated channel, Shab-related subfamily, member 1	Mm.205341	16500	-3.17	3.50
20	kif1b	Kinesin family member 1B	Mm.402393	16561	-3.44	2.22
21	kit	Kit oncogene	Mm.247073	16590	-2.59	2.01
22	lrig1	Leucine-rich repeats and immunoglobulin-like domains 1	Mm.245210	16206	-2.01	2.02
23	mal2	Mal, T cell differentiation protein 2	Mm.410994	105853	-3.11	6.49
24	mycbp	C-myc binding protein	Mm.446553	56309	-2.73	2.01
25	nat8l	N-acetyltransferase 8-like	Mm.274610	269642	-2.84	2.71
26	nmt2	N-myristoyltransferase 2	Mm.65021	18108	-2.39	3.80
27	pde4dip	Phosphodiesterase 4D interacting protein (myomegalin)	Mm.129840	83679	-6.69	2.35
28	pdgfd	Platelet-derived growth factor, D polypeptide	Mm.390122	71785	-3.74	2.49
29	pemt	Phosphatidylethanolamine N-methyltransferase	Mm.2731	18618	-2.10	2.43
30	pygl	Liver glycogen phosphorylase	Mm.256926	110095	-4.57	2.04
31	rgnef	Rho-guanine nucleotide exchange factor	Mm.252718	110596	-3.79	2.12
32	sgcg	Sarcoglycan, gamma (dystrophin-associated glycoprotein)	Mm.72173	24053	-8.37	4.75
33	sik2	Salt inducible kinase 2	Mm.104932	235344	-2.29	2.01
34	slc24a3	Solute carrier family 24 (sodium/potassium/calcium exchanger), member 3	Mm.217171	94249	-4.41	2.21
35	slc25a16	Solute carrier family 25 (mitochondrial carrier, Graves disease autoantigen), member 16	Mm.37457	73132	-3.58	2.06
36	tmem37	Transmembrane protein 37	Mm.24750	170706	-4.36	2.32
37	tmem56	Transmembrane protein 56	Mm.26088	99887	-5.70	3.93
38	trdn	Triadin	Mm.338508	76757	-16.91	12.50
39	tspan3	Tetraspanin 3	Mm.28484	56434	-2.33	2.05
40	zfhx4	Zinc finger homeodomain 4	Mm.41522	80892	-3.14	2.36
**Genes up-regulated in murine WAT while Down-regulated in human WAT**						
1	bdh1	3-hydroxybutyrate dehydrogenase, type 1	Mm.293470	71911	3.88	-2.37
2	cyfip2	Cytoplasmic FMR1 interacting protein 2	Mm.154358	76884	18.29	-4.13
3	hmgcs2	3-hydroxy-3-methylglutaryl-Coenzyme A synthase 2	Mm.289131	15360	2.11	-4.39
4	sorl1	Sortilin-related receptor, LDLR class A repeats-containing	Mm.121920	20660	8.12	-2.49

Common genes that show opposite expression in human and mouse along with absolute fold change in both organisms.

### Gene Ontology and Gene Set Enrichment Analysis

Gene Ontology of 50 differentially expressed probe sets up regulated in BAT and WAT were reviewed separately. 71 GO terms associated with BAT expression and 104 GO terms associated with WAT expression were identified. The input list was limited to 50 to identify GO terms associated with functions of the specific tissue. Of the BAT specific GO terms, lipid associated genes (GO:0015908, GO:0015245, GO:0010876, GO:0006869) were highly significant. Of the WAT specific GO terms, immune system activation associated genes such as B cell activation (GO:0042113, GO:0019724, GO:0019815, GO:0050853), T cell activation (GO:0042110, GO:0031295, GO:0030217), lymphocyte activation (GO:0046649, GO:0031294, GO:0030098, GO:0002449) and inflammatory response genes (GO:0002438, GO:0002675, GO, 0002866) were highly significant (S2 Table).

Gene set enrichment analysis on ≥2 fold change probes resulted in 52 gene sets (p≤0.1) using Pathway Studio 9.0. Adipocytokine signaling, a cell signaling pathway, had 266 measured genes including *Fabp3*, *Slc27a2*, *Rxrg* and *Aco2*. Fatty acid oxidation, a cell signaling pathway, had measured 31 genes including *Cptb1* and *Cptb2* among several which co-relates to the PPAR signaling pathway described later. Other prominent pathways identified include T-Cell NF-κβ signaling (*Cd28*, *Cd22*, *Nfkb1* and *Nfkb2*), glucose metabolism (*Fbp1*, *Pdha1*, *Eno2* and *Eno3*), tight junction assembly pathway (*Myh1*, *Myh4*, *Jam2*) (S3 Table)

### Multi-Omic pathway analysis

**Fig 2:**
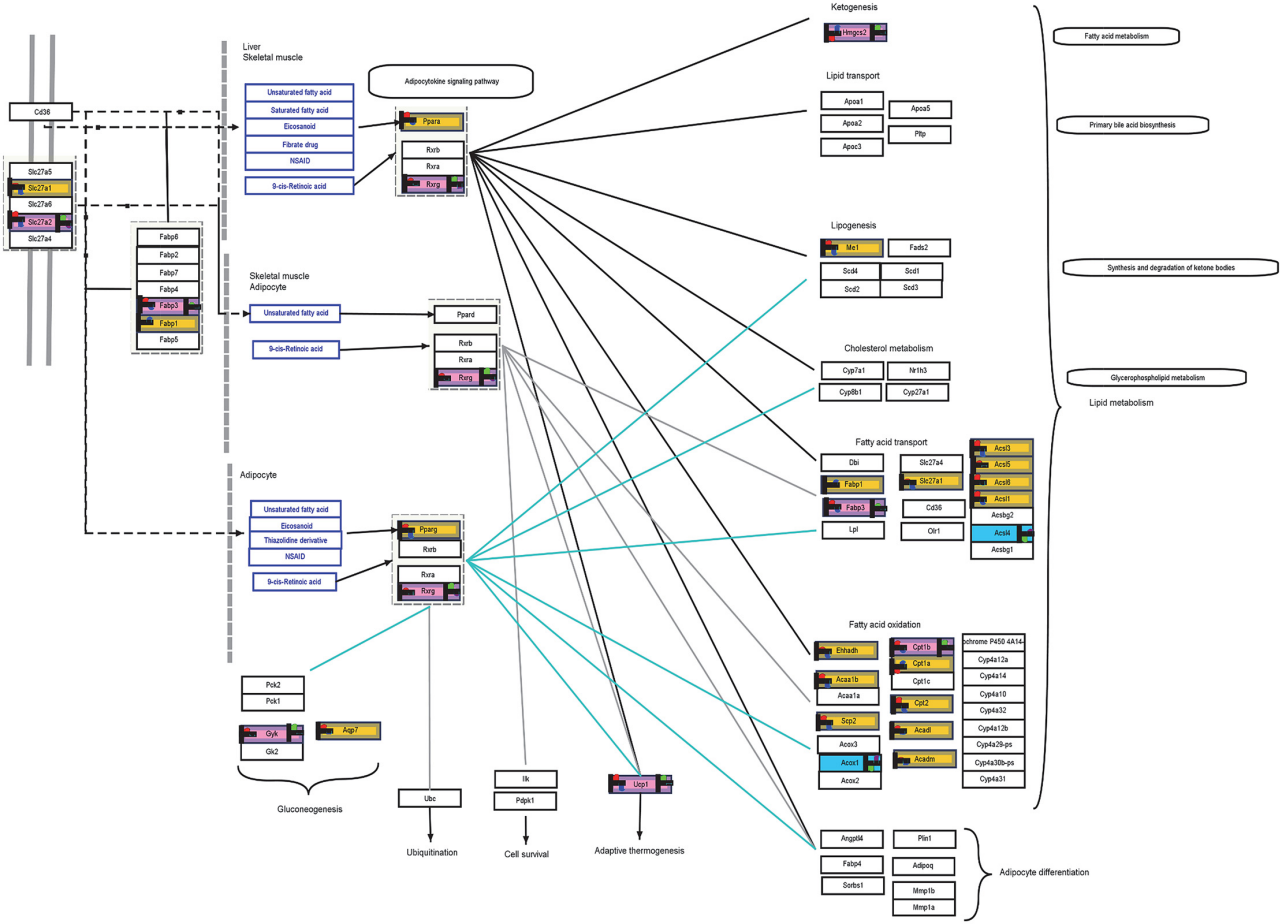
PPAR signaling pathway representing similar expression in both organisms. Yellow blocks represent expression particularly in mice while blue represent expression particular to human. Pink blocks represent expression in both organisms. The heat strips on the left of the colored bocks represent normalized signal intensity (P≤0.05, Red—expression in BAT, Blue—Expression in sWAT) in the respective tissue of LACA mouse. The heat strips on the left of the colored bocks represent normalized signal intensity (P≤0.05, Green—expression in BAT, Purple—Expression in sWAT) in the respective human tissues.

Multi-omic analysis of probe sets differentially expressed greater than 2 fold, human and mouse revealed 24 pathways common to both human and mouse models (P≤0.1, Minimum match ≥ 1). Certain genes symbols having more than one *alias* while performing same or similar function would be missed in a manual search, yet we identified based on such a pathway analysis algorithm, which took this factor into account. Some of the significant pathways common to both organisms include adipogenesis (WP447_42781), PPAR signaling pathway (WP2316_53110), Fatty acid β-Oxidation (WP1269_41314) (S4 Table). PPAR signaling is highly significant in the mouse model as they behave similar to human model in adipocytes as seen in Fig 2. *Slc27a2*, a receptor on the cell is upregulated in both mice and humans as well as *Fabp3*, a lipid transport protein. Specific to adipocytes, *Rxrg* seems to play an important role in BAT, through adaptive thermogenesis, gluconeogenesis and lipid metabolism. Genes *Fabp3*, *Cpt1b*, *Ucp1* and *Gyk* show similar expression in BAT of both organisms. Adipogenesis throws light on various genes that perform similar functions, such as growth factor *IGF1*, that contribute in differentiation of preadipocytes into mature adipocytes (S1 Fig). Interestingly, certain pathways, such as Leptin-Insulin overlap (WP578_59046) have no common genes expressing ≥2 fold change in both organisms, show possible differences in tissue level organization and expression patterns (S2 Fig).

### Multi-Array gene expression and regulation analysis

Out of the 13 BAT specific genes, 9 probe sets which represented 4 genes were identified which were highly expressed only in BAT while showing relatively low expression in all other tissues types (S3 Fig). The analysis revealed *Aco2*, *Ebf2*, *Kcnk3* and *Ucp1* are promising targets for gene manipulation in WAT to convert to BAT *(“Browning”*) having little effect on other tissues of the host.

Out of the 33 genes specific to WAT, 3 probe sets representing 3 genes were identified which were highly expressed in adipose tissue of mouse. S4 Fig reveals certain differences between other species of mice against LACA mice with genes specific to LACA WAT being expressed across adipose tissue of other species of mice.

### Multi-Array transcription factor regulation analysis

93 BAT specific factors and 213 WAT specific ones of LACA mouse were identified. Increased expression of *Zic1*, *Olig1* and *Nfe2l2* was observed in LACA mouse.

To study the expression pattern of the 93 BAT specific transcription factors above mentioned in human samples, the said genes were input into Genevestigator which generated 235 probesets for analysis, based on linear expression pattern. Since Genevestigator does not possess Human BAT data, BAT specific genes were looked for expression in WAT to identify adipose tissue preferential expression (S5 Fig).

### Validation of microarray data using quantitative PCR

**Fig 3:**
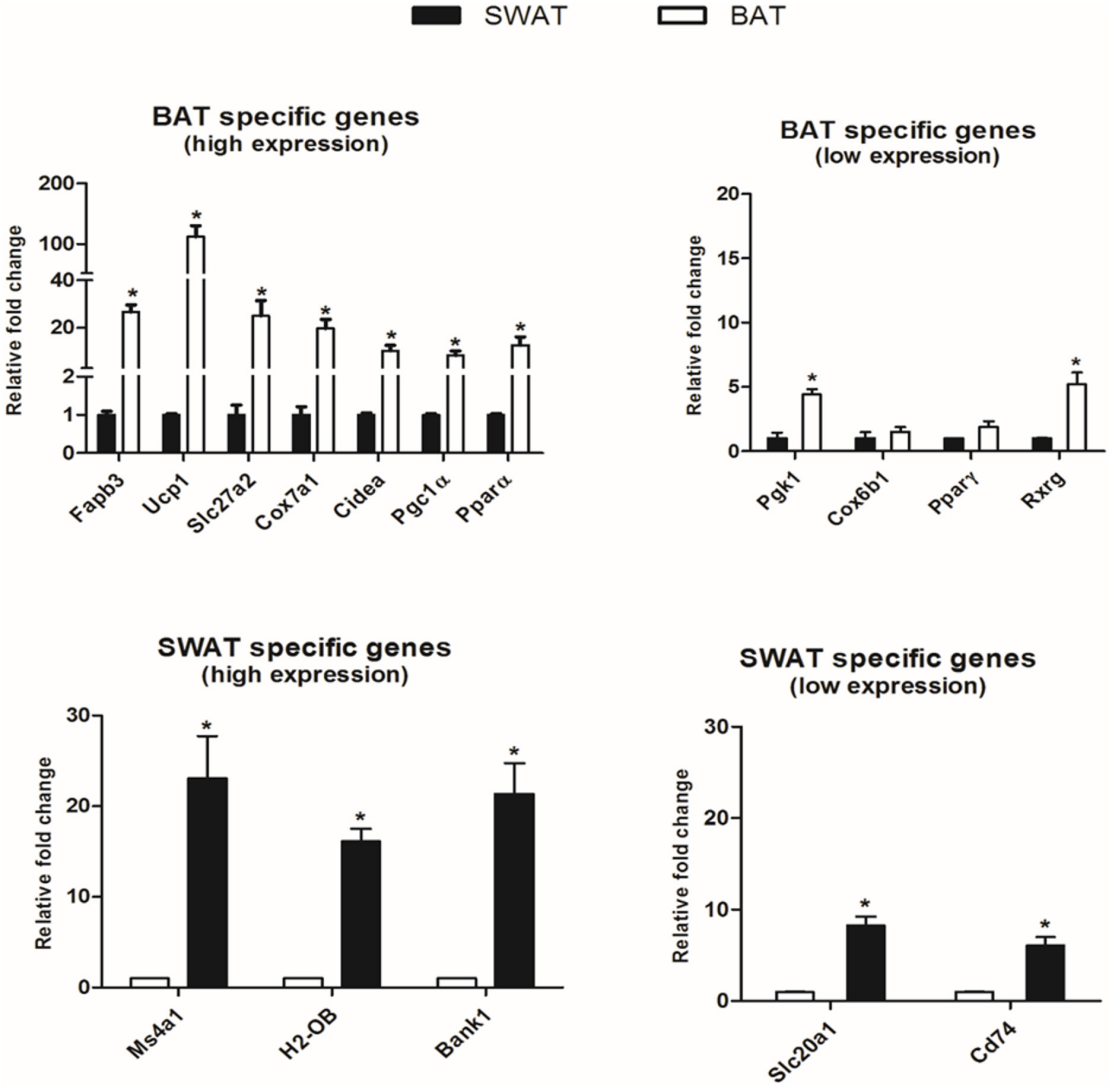
Validation of microarray data using qRT-PCR. Relative fold changes of selected genes in brown adipose tissue (BAT) against subcutaneous white adipose tissue (sWAT) validating microarray data. Fold changes represented as absolute changes of BAT against sWAT.

In order to validate the differences observed in gene expression between BAT and WAT in microarray analysis, quantitative PCR was performed to verify selected differentially expressed genes highly expressed in BAT (*Fabp3*, *Ucp1*, *Slc27a2*, *Cox7a1*, *Cidea*, *Pgc1α*, *Pparα)*, *highly expressed in sWAT (Ms4a1*, *H2-Ob*, *Bank1) moderately low expression in BAT (Pgk1*, *Cox6b1*, *Pparγ*, *Rxrg) and moderately low expression in sWAT (Slc20a1*, *Cd74)*. The qRT-PCR measurements confirmed the microarray data. Although some variation was observed in mRNA expression levels for these genes but the trend was highly consistent between the two methods, suggesting the reliability of microarray data. (Fig 3).

## Discussion

The current study establishes the differential expression of genes in BAT and sWAT of LACA mice, involvement of multiple pathways differentially expressed in these tissues while critically correlating the differential expression of highly expressed genes in mouse with humans. C57BL/6, among others, is a popular mouse model animal for high fat diet induced obesity studies [19]. On the other hand, not much has been explored about LACA mice for their potential for obesity and diabetes studies. A comparative analysis of gene expression patterns of C57BL/6 and LACA mouse revealed LACA mouse give certain advantages when studying certain pathways or phenotypic developments over C57BL/6 mice. Human adult BAT contain “Beige/Brite” phenotype while current models show classical brown phenotype [20,21]. There is a need to explore animal models that resemble human characteristics for greater relevance of therapeutic procedures in part or in whole. LACA mice can provide advantages against C57BL/6 when studying genes such as *Zic1* or *Olig1* or their associated pathways, which can be explored clinically as alternative strategies for the treatment of obesity and associated co-morbidities. Further, the study would help the scientific community identify target genes or pathways that have potential to have similar effects clinically. We believe this would save much in research costs and efforts. The study concludes with a list of genes which should or should not be targeted in murine studies in order to have greater clinical relevance.

Murine BAT and WAT showed differential expression (7003, 1176 and 401 genes showing greater than two-fold, five-fold and ten-fold change respectively) in several genes which is understandable when we look into the functional, physiological and anatomical differences between these two types of adipose tissues. Presence of multiple small fat droplets (multilocular), very dense mitochondria [22], abundant blood and nerve supply [23–25] are characteristic of BAT, differentiating it from WAT. Their thermogenic potential has been attributed to the abundance of mitochondria in these cells which uncouple fuel oxidation from ATP generation. This is caused by the presence of uncoupling protein in the inner mitochondrial membrane of these cells and results in generation of heat and expenditure of large amounts of chemical energy [26]. In our study, mitochondrial *Ucp1* was 190 times over-expressed in murine BAT as compared to sWAT. Critical analysis of our data suggests that the genes responsible for mitochondrial activity, encoding of multiple mitochondrial enzymes and other proteins are abundantly expressed in murine BAT whereas genes primarily related to tissue inflammation and immunological changes are several fold higher in sWAT suggesting that mitochondrial/metabolic processes specifically occur in BAT whereas inflammation related processes are prominent in sWAT. Our results are in accordance with earlier reports where *in-situ* microarray analysis as well as empirical qRT-PCR based studies have been used to better understand the gene profile of adipose tissues [6,27–31].

Using Gene Ontology (GeneSpring GX 12.1), GSEA (Pathway Studio Version 9.0.246.1071) and Multi-omic pathway analysis (GeneSpring GX 12.1), we have listed pathways involving differentially expressing genes in these tissues. While Gene ontology clarified GO Terms associated with BAT and sWAT of LACA mouse, reiterating sWAT was *“inflammatory and immunologically active”*, while BAT being metabolically active (S2 Table). GSEA of LACA mouse adipose tissue revealed multiple metabolism related pathways including fatty acid oxidation, glucose metabolism, Igf1r-Cebpa/Foxo1a signaling, adipocyte signaling along with NK Cell activation, various T- cell receptor signaling pathways are significantly regulated while analyzing sWAT genes with BAT genes showing 2 fold or more differential expression change in these two tissues (S3 Table). It is clearly understood here that BAT is more metabolically active than sWAT which is well supported by numerous studies over the years [32–34]. Multi-Omic comparison between LACA mouse and human adipose tissues revealed pathways common to both species, but may differ in expression profiles (S4 Table). Fig 2 represents multi-omic PPAR signaling pathway genes which showed similarities and differences between the two species. Targets such as *Slc27a1*, *Fabp3*, *Ucp1*, *Rxrg* are expressed similarly in both species while *Hmgcs2* is has an opposite expression. While Leptin-Insulin overlap pathway shown in S2 Fig shows possible mechanism of signaling in both organisms, though their expression profile describes otherwise. These results are also in accordance with existing literature[35].

We speculated that there may be significant species specific differences in mouse and human differential gene expression in BAT and WAT as observed in the multi-omic adipogenesis pathway (WP447–42781). Various factors were identified that showed differential expression in LACA mouse (*Creb1*, *Pgc1α*, *Stat1*, *Lpin1)*, while few were associated with human (S1 Fig). We decided to use data from a previously published study; analyzed and compared it with our murine data to find out the extent of species related differences [17]. The data used was submitted by Svensson and colleagues (GSE27657), which compared the microarray expression profiles from *UCP-1* positive BAT samples with *UCP-1* negative sWAT samples from same patients [17]. In our analysis we used data from this study available in public domain. We differentially compared the genes expressed in these two types of tissues and identified that there are 506 genes which are differentially expressed (≥2 fold) in these two types of tissues. It has been established for a while that the presence of metabolically active BAT in rodents has been known and its molecular characterization, genetic fingerprinting and comparative profiling with WAT in rodents is well studied. Whereas, only recently has metabolically active BAT been demonstrated in humans. It is difficult to obtain human classical BAT samples from adult humans which do not show “Brite/Beige” characteristics [21]. Murine BAT tissues, on the other hand, show classical BAT characteristics throughout their lifetime [20]. Next, we selected the 506 genes differentially expressed in human BAT and WAT and tried to find out how many were expressed similarly in mice. In total 90 genes were common in murine and human data, out of which 46 showed similar differential expression pattern. Important genes highly expressed in BAT as compared to sWAT (total 13 genes) both in mice and human are Uncoupling protein 1 (mitochondrial, proton carrier) (*UCP1)*, Fatty acid binding protein 3-muscle and heart *(FABP3)*, Creatine kinase, mitochondrial 2 (*CKMT2)*, Aconitase 2, mitochondrial *(ACO2)*, Ubiquinol-cytochrome c reductase core protein 1 (*UQCRC1)*, Pyruvate dehydrogenase e1 alpha 1 (*PDHA1)*, Cytochrome c oxidase subunit v A *(COX5A)* whereas important genes highly expressed in sWAT as compared to BAT (total 90 genes) in both mice and human are Dermatansulfateepimerase *(DSE)*, Vestigial like 3 (drosophila) (*VGLL3)*, Fibroblast growth factor 13 (*FGF13)*, Extracellular matrix protein 1 (*ECM1)*, Insulin-like growth factor 1 (*IGF1)*, Tubulin, beta 2a class ii a *(TUBB2A)*, Glypican 3 (*GPC3)* etc. Further analysis revealed that, the genes involved in mitochondrial and metabolic functions are highly expressed in BAT whereas ones which are involved in immune system and inflammatory activity are highly expressed in sWAT, which is in accordance with existing literature. More importantly, there were 44 genes which showed opposite expression in mice and human. Genes like Adrenergic receptor kinase, beta 2 (*ADRBK2)*, Phosphodiesterase 4d interacting protein (myomegalin) (*PDE4DIP)*, Potassium voltage gated channel, shab-related subfamily, member 1 (*KCNB1)*, Very low density lipoprotein receptor *(VLDLR)*, Heat shock protein family, member 7 (cardiovascular) (*HSPB7)*, Apoptosis-inducing factor, mitochondrion-associated 2 (*AIFM2)*, Carboxypeptidase m *(CPM)*, Chemokine (c-x-c motif) ligand 14 (*CXCL14)* and Flavin containing monooxygenase 1 (*FMO1)* which showed greater expression in murine BAT than sWAT but in human the trend was reversed and higher expression was seen in WAT as compared to BAT. Other genes like Forkhead box c2 (*FOXC2)*, 3-hydroxy-3-methylglutaryl-coenzyme a synthase 2 (*HMGCS2)*, Angiotensinogen (serpin peptidase inhibitor, clade a, member 8) (*AGT)*, and Sortilin-related receptor, ldlr class a repeats-containing *(SORL1)* were highly expressed in WAT as compared to BAT in mouse while the trend was opposite for human data. So, researchers should avoid using these targets or their pathways when working on understanding their role in conversion of one form of adipose tissue into other i.e. adipose tissue engineering. Particularly, the inter-species differences among important genes such as *Adrbk2*, which is known to phosphorylate agonist-occupied beta-adrenergic and *GPCRs*. Several unsuccessful experiments were carried out to activate thermogenesis in human BAT using beta3-adrenergic receptor [36–38]. Voshol et. al. reviewed various studies used *Vldlr* as a target for genetically engineered rodent models [39], but this gene shows little expression in humans. *HMGCS2*, a gene often studied in hepatic tissues, whose differential expression in human BAT were validated by Svensson et. al. while its differential expression is significantly higher in sWAT in LACA mouse. Vilà-Brau et. al. suggests the gene regulates mitochondrial fatty acid oxidation in human cell line, hep-g2 [40]. Such inter-species differences warrants more attention as they have a role in adipocyte biology and can easily draw interest of scientific community to look into them. In general, rodent studies involving these genes warrant caution as extrapolation of these to human will be questionable. When we performed group enrichment analysis on these genes, most common gene sets were G-protein coupled receptor protein signaling pathway (e.g. *Gpr34*, *Ptger3*, *Gng2*), signal transducer activity (e.g. *Ckmt2*, *Cdkn2b*, *Mobkl2b*), kinase activity (e.g. Gpr34, *Ecm1*, *Wls*), cell adhesion (e.g. *Fat1*, *Spon1*), ATP binding (e.g. *Ckmt2*, *Enpp1*), extracellular space (e.g. *Ecm1*, *Igf1*, *Spon1*).

**Fig 4:**
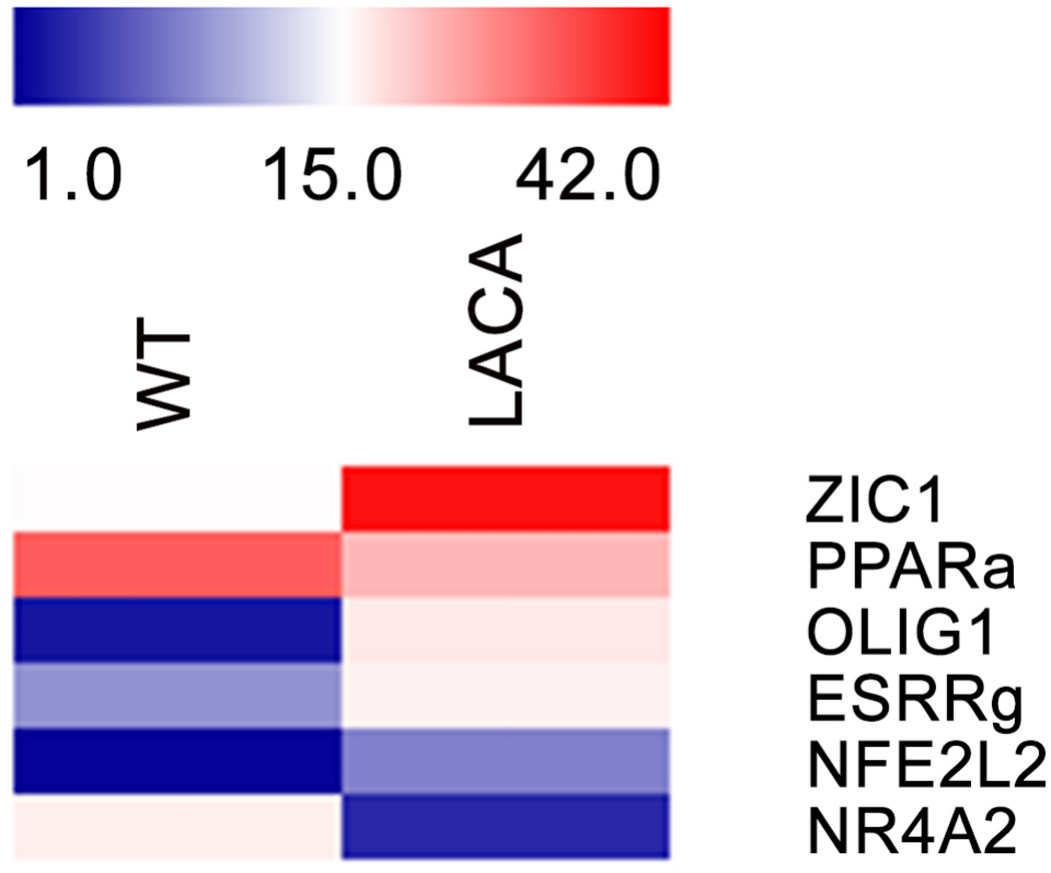
Heatmap of transcription factor expression in LACA vs C57BL/6 mice strains. Heatmap represents fold change of transcription factors with red representing fold change while blue representing low fold change. Fold change from genevestigator was calculated from their BAT/WAT ratio.

Our manual analysis co-related with the data provided by Genevestigator. A majority of gene expression in murine BAT showing opposite expression, was checked for in C57BL/6 mice and Humans. Genes such as *ACVR1C*, *ASPH*, *FMO1*, *PYGL*, *SIK2* and *SLC24A3* were highly expressed in murine BAT (S6 Fig), while their expression were up-regulated in human WAT (S7 Fig). This suggests that one needs to be cautious when targeting these genes for gene modification studies in mice as the results of animal studies may significantly differ from those of clinical trials. The variations between LACA mice and C57BL/6 (wild type) of certain significant BAT specific genes are summarized in Fig 4. Zinc family member 1 (*Zic1)* which is known to be a classical BAT marker [41] showed higher expression in LACA mouse. Oligodendrocyte transcription factor 1 (*Olig1*) whose role in obesity or adipose tissue is not well-studied, seems to play an important role in LACA mouse BAT. *Neg2l2* (Or *Nrf2*) is a proposed target for the management of obesity [42], where it is proposed to play a role in thermogenesis and prevention of lipid accumulation. Certain genetic differences between LACA mouse and other mice studied was also evident from the data, such as Nuclear Receptor Subfamily 4, Group A, Member 2 (*Nr4a2*) which is known to play a role in the regulation of thermogenesis in BAT among others [43] but is down-regulated in LACA mouse BAT suggesting an alternative mechanism for thermoregulation in LACA mouse. Several genes including *ZIC1*, *OLIG1* and *ESRRγ* showed no or low expression in human WAT forms. Certain mouse BAT specific transcription factors showed high expression in human WAT such as *EPAS1*, *THRSP*, *EBF1* and *PPARγ*. This reiterates the need for caution to be exercised when relating murine adipose tissue data with that of human.

In conclusion we have identified genes which are differentially expressed in murine BAT and sWAT of LACA and we show the inter species differences in the differential expression of genes in BAT and WAT. While we compared murine and human differential expression of two types of adipose tissues, we feel the present study is limited with respect to the number of common genes we found in two species. Multi-species studies should be carried out to understand the critical role played by various genes (their protein correlates) and their inter species differences.

## Methods

### Animals

Laca mice weighing 25 ± 3g (Male, n = 4) were procured from Central Animal House Facility of Panjab University, Chandigarh (India). Animals were housed under standard laboratory conditions (temperature 22 ± 2°C; humidity 55 ± 5%), having free access to food (Hindustan Lever Products, Kolkata, India) and water along with 12 hour light and dark cycles. The research experiments were carried out as per the guidelines set up for animal experimentation by the 'Committee for Control and Supervision of Experimentation on Animals' (CPCSEA) and INSA (Indian National Science Academy) guidelines. All the experimental procedures were approved by IAEC (Institutional Animals ethics committee, Panjab University). WAT (Subcutaneous region) and BAT (Intrascapular) of the mice were dissected from each animal and further processed.

### Microarray analysis

Total RNA was extracted from murine WAT (Subcutaneous region) and BAT (intrascapular) using ribopure RNA extraction kit (Invitrogen, USA) according to manufacturer's instructions. The quantification (total amount) and qualitative ratio metric analysis of RNA was done using Infinite M200 ProNanoQuant(Tecan, Switzerland). Integrity of the RNA samples were evaluated using 1.4% agarose gel. All RNA samples were found to be pure with no signs of degradation caused due to the extraction process. The entire microarray experiment was performed as per the protocol and guidelines provided by Affymetrix, Inc. (Santa Clara, CA). The microarray versions that were used in these studies were mouse 430.2.0 Affymetrix array. Briefly, first-strand of cDNA was generated from 500ng of RNA using a T7-linked oligo (dT) primer followed by second strand synthesis. Then, In vitro transcription was performed to synthesize aRNA incorporated with biotin-conjugated nucleotides and 15 μg of aRNA was fragmented prior to hybridization. The hybridized arrays were washed and stained with streptavidin-phycoerythrin using Affymetrix Fluidics Station 400 and finally scanned on Agilent GeneArray scanner. The microarray data is available on NCBI’s GEO database (Geo accession: GSE67389).

### Quantitative PCR analysis

Quantitative real time PCR analysis was done to validate the level of expression from microarrays for selected genes using SYBR green dye-based assay in the Applied Biosystems 7500 Fast Real-Time PCR machine as per protocol (Qiagen). Total RNA was purified as described above and 1μg of total RNA in a 20 μl reaction was reverse transcribed using single strand cDNA synthesis kit (Qiagen, USA) as per manufacturer’s instructions. Relative expression of selected genes such as *Fabp3*, *Slc27a2*, *Cox7a1*, *Ms4a1*, *H2-Ob*, *Bank1*, *CIDEA*, *FABP3*, *RxRg* was determined by quantitative PCR (qPCR). *Additionally Ucp1*, *Pparα*, *Ppargc1α (Pgc1α)*, *Pparγ* was determined by quantitative PCR (qPCR) using custom designed PCR Array (PAMM 049A, Standard Mouse RT2 profile PCR Array, SABiosciences, Qiagen, USA). The conditions for RT-PCR were: 95°C for 10 minutes followed by 40 cycles at 95°C and held at 60°C for 1 minute. All measurements were performed in duplicate. Gene expression was normalized by the mean of two genes, *i*.*e*. GAPDH and β-Actin while GAPDH and HSP90AB was used in the custom array for murine white and brown adipose tissues. Since the expression of these genes remain unchanged in BAT and WAT they were chosen as housekeeping gene (gene based selection). Data was analyzed as per protocols for ΔΔCt method. Custom array data was analyzed using web based software provided by SABiosciences, USA.

### Microarray Data mining

DNA microarray data was analyzed using GeneSpring GX 12.1 software (Agilent Technologies) with the raw data files (.cel) of the Affymetrix Mouse chip imported into the software. RAM (Robust Multichip Averaging) algorithm was used to normalize the data where it was subjected to quantile normalization with median of all samples taken for baseline transformation. Gene expression profiles of murine WAT and BAT were studied by generating a differential expression list. The two parameters were subjected to paired student's T test statistical analysis where the false-positive event (p-value cut-off = 0.05) calculation done using asymptotic method on the normalized data assuming equal variances, incorporating Benjamini and Hochberg false discovery rate multiple-testing correction set at a rate of 0.05. The resultant list of genes differentially expressed between the two parameters were filtered to include only those that expressed absolute fold change by greater than two-fold, five-fold and ten-fold change.

### Comparison of mouse data with human data

In order to identify the variation between human and mouse genes differentially expressed in WAT and BAT, the publicly available microarray data (GEO dataset GSE27657) of human WAT (subcutaneous) and BAT (peri-thyroid region) was first analyzed using GeneSpring GX 12.1 software (Agilent Technologies) in a similar way as mentioned above. Then, the number of differentially expressed genes in human WAT vs. BAT with greater than two fold change expression was compared with our data *i*.*e*. with genes differentially expressed in murine WAT and BAT manually (using gene symbols as reference) and using multi-omic pathway analysis (p ≤ 0.1, minimum match ≥1). Venn diagram was generated to highlight similarities and differences between the gene expression profiles of two species.

### Pathway Mapping and Enrichment Analysis

Gene ontogeny was performed using GeneSpring 12.1 using top 50 individually expressed probe sets of murine BAT and WAT (differential expression fold change 20 ± 2) to study GO terms associated with BAT or sWAT specific tissues (Corrected P value ≤ 0.1).

Gene set enrichment analysis and functional annotation of genes was carried out using Pathway Studio software version 9.0.246.1071 on ResNet 9.0 Mammal database. Differentially expressed genes were mapped on available pathways in Pathway Studio. Pathways enriched with (List 1: LACA mouse sWAT vs. BAT ≥2 fold regulated genes; List 2: Human WAT vs. BAT ≥2 fold regulated genes) genes were identified by enrichment algorithm which deploys Fischer Exact test (P ≤ 0.1). An error margin of 10% was taken to include well known and documented genes in the gene sets.

### Multi-Array gene expression analysis

Genes expressed in LACA mouse were investigated for their expression pattern across various publically available mouse microarray data using Genevestigator online tool using respective Affymetrix platforms (Mouse 430 2.0 Array, Human 133 Plus 2.0 Array)[44]. Genevestigator uses RMA for normalization with P≤0.1. Genes that were common to LACA mouse and Human data were first investigated for comparing similarities and differences between LACA and other mice studied upon from an obesity oriented perspective. The BAT or WAT specific list was input in Genevestigator, and the expression of these genes in known mouse models were looked into. Using linear and log_2_ normalized expression, heatmaps and BAT/WAT relative fold expression in the data sets were analyzed and compared with LACA mouse data.

To study the expression pattern of transcription factors in adipose tissue, probesets expressed ≥2 fold were compared with the factors listed in the MTFDB (http://genome.gsc.riken.jp/TFdb/). Transcri≥tion factors in LACA mouse data was identified and compared using Genevestigator online tool. Heatmap was made using MeV software tool.

### Statistical analysis

The differences in expression of genes between BAT and WAT were analyzed by paired student's t-test followed by Benjamini and Hochberg FDR corrections. P-values of ≤0.05 were considered statistically significant. GSEA and Multi-omic analysis was conducted at P-value cut off of 0.1 using default parameters. qRT-PCR data was analyzed using One-tailed unpaired t-test (Welch’s Correction) (P ≤ 0.05) with Prism Graphpad.

## Supporting Information

S1 Fig Adipogenesis specific genes expressed in both organisms. Multi-omic pathway analysis (P≤0.1, minimum match ≥ 1) of adipose tissue specific genes expressed in both organisms revealed various mouse specific and human specific genes with some common to both organisms. Genes marked in yellow are expressed in mouse, while genes marked blue are expressed in humans and those marked pink are expressed in both organisms. Heat-strips having Red-Blue (Red—BAT, Blue—WAT) colours represent normalized signal intensities in mouse. Heat-strips having Green-Purple (Green—BAT, Purple—WAT) colours represent normalized signal intensities in humans.
(TIF)

S2 Fig Leptin-Insulin Overlap genes having no common expression. Multi-omic pathway analysis (P≤0.1, minimum match ≥ 1) of WAT showed genes expressed in Leptin signalling involved in mice that were significantly upregulated predominantly in WAT of both organisms, represented by trailing heat-strips (Red—BAT, Blue WAT). While yellow boxed genes represent expression in LACA mouse, Blue boxed genes expression in Humans.
(TIF)

S3 Fig BAT specific expression analyzed across tissues of mice. 13 genes that were highly expressed in LACA mouse BAT were looked into for expression pattern in C57BL/6 mice. 9 probe sets representing 4 genes (P≤0.1) were identified in the public mouse data available on Genevestigator using default parameters of log expression. The expression heatmap was organized based on highest to lowest *UCP1* expression in various tissues, with red representing maximum expression while white representing minimum.
(TIF)

S4 Fig WAT specific expression analyzed across tissues of mice. 13 genes that were highly expressed in LACA mouse WAT were looked into for expression pattern in C57BL/6 mice. 3 probe-sets representing 3 genes (P≤0.1) were identified using Genevestigator using default parameters of log expression. The expression heatmap was organized based on highest to lowest *Sik2* expression in various tissues, with red representing maximum expression while white representing minimum.
(TIF)

S5 Fig BAT specific transcription factor expression analyzed across tissues of mice. 93 BAT transcription factors (represented by 132 probesets of human counterparts) that were highly expressed in LACA mouse BAT were looked into for expression pattern in human samples across 151 anatomical tissues. 235 probe-sets representing (P≤0.1) were identified using Genevestigator using default parameters of linear expression. The expression heatmap was organized based on highest to lowest *EPAS1* expression in various tissues, with red representing maximum expression while white representing minimum.
(TIF)

S6 Fig BAT specific gene expression showing opposite expression in C57BL/6. LACA mouse BAT specific genes that showed opposite expression in human was checked for their expression pattern in C57BL/6 using Genevestigator. 40 genes that showed opposite expression in LACA BAT.
(TIF)

S7 Fig Murine BAT specific genes in Human WAT showing opposite expression. LACA mouse BAT specific genes that showed opposite expression in humans, thus expressed in their WAT (SAAT, SAT and MAT). 40 Murine BAT gene names analyzed resulted in 23 Human probe sets, which were compared against 151 anatomical parts available on Genevestigator database using default parameters of linear expression. The expression heatmap was organized based on highest to lowest *ACVR1C* expression in various tissues, with red representing maximum while white representing minimum.
(TIF)

S1 Table List of probe sets showing ≥2 fold change in LACA sWAT and BAT. Probe sets differentially expressed ≥2 fold change in sWAT are expressed as positive fold change while those expressed ≥2 fold change in BAT are expressed as negative fold change. The table also provides additional information provided by GeneSpring GX 12.1 regarding each of the probe sets such as Gene symbol, Entrez gene id and GO category.
(XLSX)

S2 Table List of Gene ontology terms associated with top 50 differentially expressed BAT and sWAT probe sets. The table contains associated p-values for each of the lists for each of the terms.
(XLSX)

S3 Table List of gene sets enriched after analyzing the ≥2 fold differentially expressed probe sets. The p value cut-off for the list generated was set at 0.1 for selectivity of gene sets which are highly functional in either of the tissues.
(XLSX)

S4 Table List of pathways identified in multi-omic analysis between LACA mouse and Humans. The table lists common pathways of differentially expressed probe sets of human and mouse at p <0.1, with minimum match of ≥1.
(XLSX)
