# Supplementary figures and images for: Microarray Based Gene Expression Analysis of Murine Brown and Subcutaneous Adipose Tissue: Significance with Human
Source: PLoS One. 2015 May 26;10(5):e0127701. doi: 10.1371/journal.pone.0127701 (PMC4444008; doi:10.1371/journal.pone.0127701)

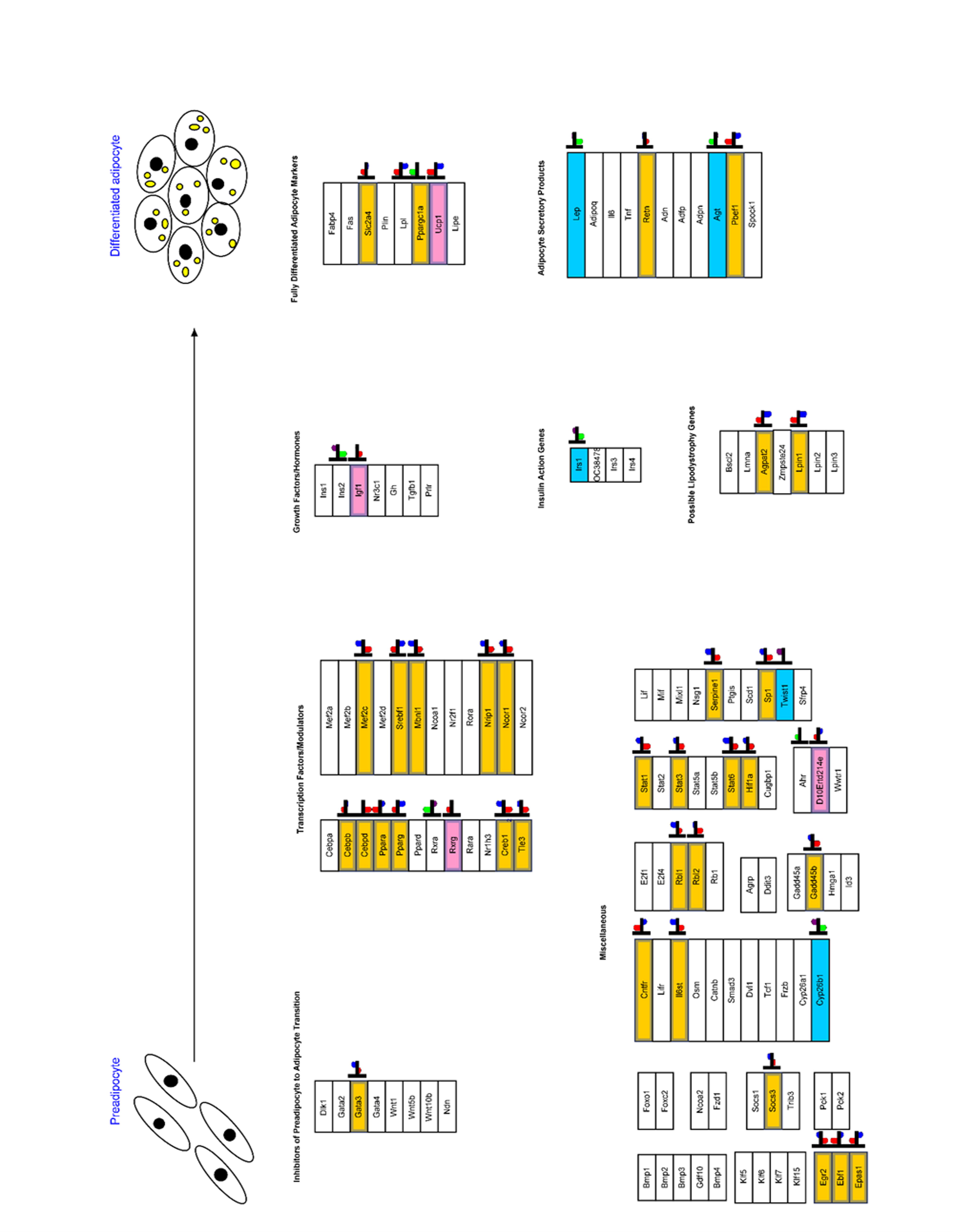

Supplement: S1 Fig — Multi-omic pathway analysis (P≤0.1, minimum match ≥ 1) of adipose tissue specific genes expressed in both organisms revealed various mouse specific and human specific genes with some common to both organisms. Genes marked in yellow are expressed in mouse, while genes marked blue are expressed in humans and those marked pink are expressed in both organisms. Heat-strips having Red-Blue (Red—BAT, Blue—WAT) colours represent normalized signal intensities in mouse. Heat-strips having Green-Purple (Green—BAT, Purple—WAT) colours represent normalized signal intensities in humans. (TIF) [file pone.0127701.s001.tif]

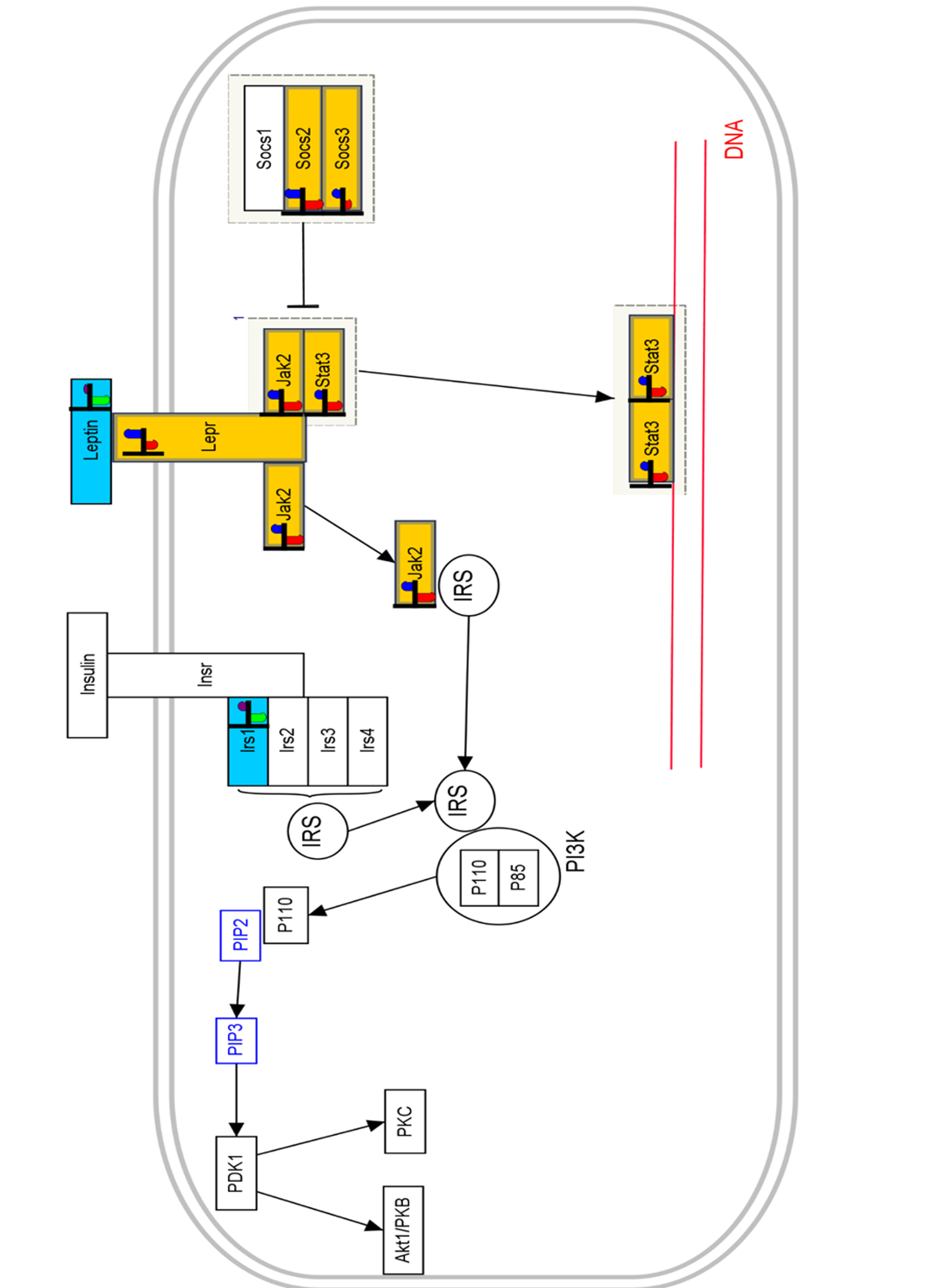

Supplement: S2 Fig — Multi-omic pathway analysis (P≤0.1, minimum match ≥ 1) of WAT showed genes expressed in Leptin signalling involved in mice that were significantly upregulated predominantly in WAT of both organisms, represented by trailing heat-strips (Red—BAT, Blue WAT). While yellow boxed genes represent expression in LACA mouse, Blue boxed genes expression in Humans. (TIF) [file pone.0127701.s002.tif]
